# Genomic Analysis of the Giant Red Shrimp (*Aristaeomorpha foliacea*) Using Next-Generation Sequencing: Set of Tools for Population Studies

**DOI:** 10.3390/genes15111360

**Published:** 2024-10-23

**Authors:** Sandra Heras, Alba Abras, Aleix Palahí, Jose-Luis García-Marín, María Inés Roldán

**Affiliations:** 1 Laboratori d’Ictiologia Genètica, Universitat de Girona, ES-17003 Girona, Spain; sandra.heras@udg.edu (S.H.); aleix.palahi@ebc.uu.se (A.P.); joseluis.garcia@udg.edu (J.-L.G.-M.); marina.roldan@udg.edu (M.I.R.); 2 Department of Ecology and Genetics (IEG), Uppsala University, Norbyvägen 18D, SE-752 36 Uppsala, Sweden

**Keywords:** *Aristaeomorpha foliacea*, next-generation sequencing, microsatellite loci, parentage studies, population genetics, giant red shrimp

## Abstract

Background/Objectives: The giant red shrimp, *Aristaeomorpha foliacea*, is a valuable marine fishing resource. The conservation of species, especially exploited ones, depends on a good knowledge of their biology, as well as the development of appropriate management plans based on the identification of genetically differentiated units or genetic stocks. Microsatellites are widely used molecular markers to detect genetic stocks in penaeoid shrimps and prawns. This study aimed to develop and characterize new microsatellites for *A. foliacea*. Methods: Next-generation sequencing based on 454 pyrosequencing revealed 58 candidate microsatellite loci for *A. foliacea*. These were tested on a panel of 8 individuals representative of its worldwide geographical distribution, and 19 polymorphic loci were identified and subsequently validated and characterized in 30 individuals from a single population in the Mediterranean Sea. Results: As a result, 10 polymorphic loci were identified, which did not present linkage disequilibrium and showed a range of alleles per locus and an observed and expected heterozygosity of 2–10, 0.0667–0.5567, and 0.0661–0.8511, respectively. Nine out of these loci were under Hardy–Weinberg equilibrium and showed a combined exclusion probability of 0.9202 and 0.9968 in parentage and identity analysis, respectively. Conclusions: This set of loci will provide a strong set of tools to (i) perform parentage studies and (ii) examine connectivity patterns (horizontal and vertical), including examining the population structure of this species at a variety of geographical scales and, particularly, between exploited populations in shallow waters and deeper unexploited populations.

## 1. Introduction

The giant red shrimp, *Aristaeomorpha foliacea* (Risso, 1827), is a marine decapod belonging to the family Aristeidae (superfamily Penaeoidea) [1]. Adult individuals are large-sized, with the most common range of 13–14 cm in males and 17–20 cm in females, and present a red color with darker violet reflections [2]. It is a demersal species that inhabits the muddy bottoms of the continental slope, often associated with submarine canyons [3], and has a bathymetric range from 123 m to 1145 m depth [4,5] with a higher density of individuals in the depth range of 400–800 m [5,6]. This shrimp is distributed in the Mediterranean Sea and in the Atlantic, Indian, and West Pacific Oceans [2,7,8]. Within the Mediterranean Sea, the distribution of *A. foliacea* is patchy and shows an eastward increase in abundance [9,10].

The giant red shrimp is one of the most significant economic resources in the Mediterranean region [11], ever since its fishing began in the 1930s in the Ligurian Sea [6]. Currently, the species has a global capture production of approximately 2300 tons per year [12], and it holds a high commercial value in the markets (EUR 50–100/Kg). In addition, since the late 1980s, fisheries have also been developed off the Australian coast [13] and, later, in the Mozambique Channel [14]. Brazil began fishing in the early 2000s [15], and more recently, new fishing grounds have been discovered in Mexico [16] and Colombia [17]; however, direct fishery has not yet been established in these latter regions.

The Scientific Advising Committee (SAC) of the General Fisheries Commission for the Mediterranean Sea (GFCM), which is part of the Food and Agriculture Organization (FAO), included *A. foliacea* in the list of priority species for fishery assessment, noting that the development, conservation, and management of this marine resource should be promoted [18]. Although some EU Mediterranean countries (France, Italy, and Spain) have separately adopted some management plans for their regional giant red shrimp fisheries, the lack of coordinated action between these countries has reduced their effectiveness [19]. In both the Western Mediterranean (GSAs 9–11) and the Central–Eastern Mediterranean (GSAs 12–16, 18–27), the SAC has recently reported that *A. foliacea* populations are in a poor state due to overfishing and that the current level of exploitation of these populations may not be sustainable [12,20,21]. In addition, the Scientific Technical and Economic Committee for Fisheries (STECFS) of the European Commission has warned that the biomass of the giant red shrimp is decreasing in the Mediterranean (GSAs 8–11), and, consequently, management measures must be taken [22].

Given the significant commercial value of the giant red shrimp in the Mediterranean, several studies have been conducted, mainly in the field of fisheries biology, to analyze its distribution, abundance, and biomass [5,9,10,23,24,25,26], its ecology [3,27], its reproductive biology [28,29], and its fishing viability [30,31]. However, the arguably most important and challenging aspect for the successful implementation of a comprehensive management plan for a fishery is the identification of its most appropriate biological management units, which the SAC has recently recommended for *A. foliacea* [21]. According to the FAO [32], genetic diversity among and within natural populations should serve to recognize these management units in the so-called genetic stocks. The genetic diversity of the different stocks of the exploited species may be reduced as a result of the pressure of overfishing, and thus, their ability to adapt and survive environmental changes might be increasingly compromised [33]. Therefore, it is crucial and urgent to implement a management plan for *A. foliacea* fisheries that focuses on the identification and conservation of its genetic stocks. The disappearance of regional habitat-adapted fisheries can be the onset of this species extinction [33].

The first genetic markers used in studying *A. foliacea* were the Inter Simple Sequence Repeats (DNA-ISSR) [34]. However, these markers were extremely variable, causing saturation and making them less sensitive and unable to detect any genetic structuring in the species. Consequently, they were not used in successive studies. Later, the mitochondrial DNA marker cytochrome oxidase subunit I (COI) was used, showing differences between populations on a large geographical scale and identifying three major lineages distributed on the Mediterranean Sea, Mozambique, and Australia [35]. These findings were also supported by the nuclear markers phosphoenolpyruvate carboxykinase (PEPCK) and sodium–potassium ATPase α-subunit (NAK), although the differences were subtler [36]. Furthermore, these studies suggested a scenario of cryptic speciation, where the Australian lineage would be a genetically distinct species [35,36]. In addition, Fernández et al. [35] also reported significant genetic differences among samples across the Mediterranean Sea. However, none of these markers were able to identify distinct genetic lineages due to a lack of statistical significance in comparisons between samples collected in the Western and Eastern Mediterranean.

Given the previous studies, the necessity remains of developing alternative molecular markers that exhibit sufficient variability, sensitivity, and resolution to be able to identify the potential genetic stocks at a local or regional level in *A. foliacea*; microsatellites, also known as Simple Sequence Repeats (SSRs), are the frontrunner candidates. Microsatellites have been widely used for population structure analysis, stock identification, and effective population size estimation in fisheries management and conservation genetics of exploited aquatic animals [37,38,39,40]. In particular, they have proven useful in identifying genetic stocks of penaeoid shrimps and prawns worldwide [41,42,43,44,45,46,47]. In addition, they are superior for parentage analysis and are more cost-effective for genotyping compared to other molecular markers, such as Single Nucleotide Polymorphisms [38,40].

So far, only nine polymorphic microsatellite loci of *A. foliacea* have been reported by Cannas et al. [48] using the FIASCO protocol [49]. Out of these, only five were in Hardy–Weinberg equilibrium (HWE) and did not exhibit linkage disequilibrium. However, these polymorphic loci failed to detect significant genetic differentiation among the studied Italian fishing grounds of Sardinia and Sicily [50]. The development of microsatellites using Next-generation sequencing (NGS) based on the 454 pyrosequencing is a fast and efficient approach [51]. Due to its effectiveness, this technology has become the preferred choice for developing microsatellites in non-model organisms [52]. It has been successfully employed to obtain useful SSRs in other species of Aristeidae [53], as well as in other penaeoids [54,55,56] and decapods [57,58,59,60,61,62,63,64].

Considering all the above relevant reasons, this study aims to develop and characterize new and necessary microsatellites for *A. foliacea* using NGS based on 454 pyrosequencing. These molecular markers could assist in identifying potential genetic stocks in *A. foliacea* and their connectivity at a local geographic scale, as well as studying its reproductive patterns through parentage analysis. Integrating these genetic data into the biological knowledge of the species will provide valuable information for implementing comprehensive management policies for its fisheries, thereby ensuring its long-term preservation.

## 2. Materials and Methods

### 2.1. DNA Extraction and Next-Generation Sequencing

The total genomic DNA was isolated from a portion of about 10 mg of abdominal muscle tissue of one giant red shrimp individual caught by bottom trawl and provided by the *Instituto Español de Oceanografia* from Balearic Islands (Cabrera: 39°02′ N, 02°39′ E) (specimen-voucher: LIG Af329, stored at our laboratory) following a phenol–chloroform method with minor modifications [53,65]. DNA integrity was checked on a 0.8% agarose gel with ethidium bromide (0.5 mg/mL), and the total yield was quantified with both a NanoDrop ND-1000 spectrophotometer (Thermo Fisher Scientific, Waltham, MA, USA) (100 ng/μL) and Picogreen reagent (Thermo Fisher Scientific) (110 ng/μL). NGS was performed in 2012 on a Roche 454 GS Junior Pyrosequencer by Pompeu Fabra University’s Genomics Service (Barcelona, Spain), and the produced reads were assembled into contigs with GS De Novo Assembler program (Roche 454 Life Sciences 2006–2012) using the default settings with two modifications (heterozygoteMode: true; largeGenome: true), as outlined by Heras et al. [53]. Contigs of less than 100 pb were discarded.

### 2.2. Microsatellite Loci Screening

Sequences containing putative microsatellite loci were filtered from contigs using Tandem Repeats Finder v. 4.09 [66]. Match, mismatch, and indel alignment parameters were set at 2, 7, and 7, respectively; the minimum alignment score to report repeats was set to 30, the maximum period size was set to 5 (penta-nucleotides), and the option of recording 500 flanking nucleotides on each side of repeats was selected. Repeat motifs with a period size of only one (mono-nucleotides) were discarded, as well as the number of repeats outside the range of 5–50. Then, those candidate loci with at least 30 bp on each flanking side were selected to allow primer design with Primer 3 v. 0.4.0 [67]. Product size ranges were set at 50–450 bp, and adjacent repetitions separated < 100 bp were considered the same locus (compound microsatellite). Primer specificity was verified using NCBI’s Primer-Blast tool to ensure that primers hybridize only within their designed contig region. In addition, our contigs were used as query sequences in NCBI’s BLASTn search, with a threshold of e-values < 10^–10^ to exclude microsatellites previously reported by Cannas et al. [48].

### 2.3. Verification of Candidate Microsatellite Loci

The candidate microsatellite loci were preliminarily tested at our laboratory to ensure correct amplification and polymorphism in four distant fishing areas by genotyping a panel of eight individuals of *A. foliacea* collected by bottom trawl from the Eastern Mediterranean Sea (Ionian Sea, *n* = 1 and Aegean Sea, *n* = 1) by the Hellenic Centre for Marine Research, and from the Western Mediterranean Sea (Cabrera, *n* = 2), the Atlantic Ocean (Gulf of Cadiz, *n* = 2), and the Indian Ocean (Mozambique Channel, *n* = 2) by various surveys conducted by the *Instituto Español de Oceanografía* in 2008 (see details Fernández et al. [35]).

The phenol–chloroform method was used to extract DNA from each individual as previously described [53,65]. DNA quantity, quality, and integrity were assessed by a NanoDrop ND-1000 spectrophotometer (Thermo Fisher Scientific) and 1.5% agarose gel electrophoresis with GelRed^®^ (0.5 mg/mL) (Biotium, Fremont, CA, USA). The DNA was then diluted to an optimal concentration of 40 ng/μL for further PCRs.

We used a three-primers-nested PCR approach developed by Schuelke [68] to amplify the DNA. In this method, a specific non-labeled forward primer with a universal 5′M13 tail (5′-CACGACGTTGTAAAACGAC-3′) and a specific non-labeled reverse primer were used for each microsatellite locus. The third primer was the M13 labeled with 6-FAM and was used as a common forward primer for all loci amplification to reduce genotyping costs.

Amplification for each putative microsatellite loci was conducted in a 21 µL total reaction mixture containing 12.5 µL of ddH_2_O, 2 µL of 10X NH4 reaction buffer, 1 µL of MgCl_2_ (50 mM), 2 µL of dNTP (10 mM), 0.04 µM of the specific forward primer, 0.2 µM of both specific reverse primer and forward 6-FAM-labeled universal M13 primer, 0.5 U of BIOTAQTM DNA polymerase (Bioline, Meridian Bioscience, London, UK), and 1 µL of DNA template (~40 ng).

Thermal PCR cycling conditions were an initial denaturation step at 94 °C for 2 min, followed by 35 cycles of denaturation at 94 °C for 30 s, annealing at the optimal temperature (see Table 1) for 90 s, and extension at 72 °C for 90 s; followed by a final extension step at 72 °C for 10 min.

The success of amplification for each potential microsatellite locus was verified on a 2% agarose gel with GelRed^®^ (0.5 mg/mL). Then, 1 µL of PCR product was mixed with 10 µL of Hi-DiTM formamide and 0.1 µL of GeneScanTM 500 LIZ size standard and loaded into an ABI PRISM 3130 Genetic Analyzer (Applied Biosystems). Finally, allele scoring was manually checked using Geneious v.7.1.9 [69], and polymorphism was assessed across the eight representative individuals of *A. foliacea*.

### 2.4. Data Analysis for Polymorphic Microsatellite Loci Validation

We genotyped 30 individuals from Cabrera (Western Mediterranean Sea), where the NGS individual was collected, to assess the usefulness of polymorphic microsatellite loci for future population genetic studies of *A. foliacea*.

DNA extraction, PCR reactions, and genotyping of these additional *A. foliacea* specimens were performed following the same conditions as in the verification phase but with the use of supplementary forward M13 primers labeled with other fluorescent dyes (VIC, NED, and PET). This method allows the simultaneous genotyping of multiple singleplex PCR products for different microsatellite loci with a similar allele size range. These singleplex PCR product mixtures were loaded onto an ABI PRISM 3130 Genetic Analyzer in two separate sets of 4 and 6 best candidate loci obtained in the verification phase.

To validate the usefulness of the polymorphisms at these 10 microsatellite loci for population studies on *A. foliacea*, we estimated the observed and expected heterozygosities (*H*_o_ and *H*_e_, respectively), the inbreeding coefficient (*F*_IS_) using allele identity, and tested for linkage and HWE using Genepop v.4.7 [70]. Test significances in Genepop were assessed using an exact test (Markov chain Monte Carlo method with 10,000 dememorizations, 20 batches, and 5000 iterations per batch), and Fisher’s method was used for a global test of HWE. Micro-Checker v.2.2.3 [71] with 1000 Monte Carlo bootstrap randomizations was utilized to assess the presence of null alleles as well as scoring errors, such as stuttering or allele dropout, in the loci that displayed significant deviation from HWE. Significance values for all multiple tests were adjusted by applying Bonferroni correction. Finally, Cervus v.3.0.7 was used to determine the suitability of loci for parentage and identity analyses by calculating combined non-exclusion probabilities over loci [72].

## 3. Results and Discussion

### 3.1. NGS De Novo Assembly and Putative Microsatellite Loci Detection

The NGS of *A. foliacea* generated a total of 39,088,042 bp (confidence quality mean = 29.1; at least Q40 = 24.2%), distributed among 124,155 reads with an average length of 315 bp. These values were slightly lower than those obtained for another Aristeidae shrimp, *Aristeus antennatus*, using the same 454 methodology (56,772,861 bp, 165,507 reads, and 343 bp of average length; [53]). However, they were consistent with those for other decapods (31,344,455–55,152,741 bp, 103,385–186,890 reads, and 245–375 of average length) [54,57,59,62,73].

A total of 13,213 reads of *A. foliacea* were assembled into 1973 contigs (917,258 bp) with a range from 100 to 13,843 bp and an N50 contig size of 889 bp. These results were very similar to those obtained for *A. antennatus* with 10,613 reads assembled into 2029 contigs, covering 895,246 bp, ranging from 100 to 12,986 bp and an N50 contig size of 858 bp [53].

Overall, 232 tandem repeats were detected for the giant red shrimp, distributed across 167 contigs. Out of these repeats, 191 had a minimum copy number of five and exhibited di- (45%), tri- (36%), tetra- (13%), and penta-nucleotides (6%) as repeat motifs. The number of tandem repeats previously detected in *A. antennatus* (317) was higher (about 1.4 more repeats per bp) than in *A. foliacea* [53]. On the other hand, both species had di-nucleotides as the most common repetitions, as occurs in other commercially valuable decapod shrimps [74,75,76].

A total of 74 putative microsatellite loci presented at least 30 bp at each flanking region, but Primer3 designed primers for 58 of them after Primer-Blast specificity filtering. Although the number of candidate loci retained to be later verified by PCR was lower compared to *A. antennatus* (97; as reported by Heras et al. [53]), it is noteworthy that the primers for *A. antennatus* were not filtered for specificity with Primer-Blast. In addition, our value falls within the range observed in other studies using 454 technology to identify microsatellite loci in crustaceans, including decapods (25–1420 microsatellite loci identified after filtering and primer design; [73]).

Finally, the BLASTn search in GenBank did not yield matches with previously developed microsatellites for *A. foliacea* [48].

### 3.2. Verification of Candidate Microsatellite Loci

A total of 29 out of 58 candidate microsatellite loci were successfully amplified in the eight representative individuals of *A. foliacea* from distant fisheries. Among these, 19 were found to be polymorphic, with 2 to 6 alleles per locus and an average of 3.1 (Table 1). This resulted in a success rate of 33%, which is again comparable to the one previously reported for *A. antennatus* (36%, as reported by Heras et al. [53]). These results are consistent with those observed in other decapods using the 454 system (16–50% success rate) [56,57,58,59,61,63,64].

### 3.3. Validation and Characterization of Polymorphic Microsatellite Loci

The validation of the 19 polymorphic loci by genotyping 30 individuals from Cabrera resulted in the discarding of 7 loci due to ambiguous multiple peak scores in some individuals. Ten out of the remaining twelve loci were polymorphic in the analyzed sample and presented a clear peak pattern for genotyping, yielding a mean number of alleles per locus of 3.8 (2–10) and a mean *H*_o_ and *H*_e_ of 0.276 (0.067–0.557) and 0.347 (0.066–0.851), respectively. Cannas et al. [48] obtained a similar number of polymorphic loci (9) for *A. foliacea* following the FIASCO protocol [49], but the mean number of alleles per locus (8.9) and the mean *H*_o_ (0.707) and *H*_e_ were higher than those displayed in our study. However, our values fell within the range observed for *A. antennatus* (*N*_A_ = 2–14; *H*_o_ = 0.000–1.000; *H*_e_ = 0.050–0.968) [53] as well as for other shrimps and prawns in the Penaeoidea superfamily (*N*_A_ = 2–24; *H*_o_ = 0.048–1.000; *H*_e_ = 0.048–0.940) [44,46,47,56,77].

Of the 10 polymorphic loci analyzed in the Cabrera sample, the genotype proportions of nine of them follow expectations under HWE using the Bonferroni adjustment for significance level (*p* = 0.005). The mean number of alleles per locus was 3.1, and the mean *H*_o_ and *H*_e_ were 0.258 and 0.290, respectively (Table 2). Overall, for the loci, this Cabrera sample showed no significant deviation from HWE (*p* = 0.152), and linkage disequilibrium between loci was not detected after applying the Bonferroni correction (*p* = 0.001). The study conducted by Cannas et al. [48] found that only seven out of their nine polymorphic loci were in HWE, and one pair of loci were in linkage disequilibrium. However, these loci have not proven to be useful for population studies of the giant red shrimp [50].

The sole locus in our study that deviated significantly from the HWE expectations (Af40b) displayed both stuttering—potentially leading to genotyping errors—and a null allele frequency of 0.217, which is indicative of a heterozygote deficit and a subsequent statistically significant and positive F_IS_ value (Table 2). Previous microsatellite studies in Aristeidae and Penaeoidea shrimps have also found loci under significant departures from HWE due to heterozygote deficiency [44,46,53] and references therein [56,78]. Furthermore, some of these studies also reported that disequilibria could result from the possible existence of null alleles [46,53] and references therein [56,78]. In the end, the nine polymorphic loci under HWE presented in our study exhibited a combined exclusion probability of 0.9202 in parentage analysis when the parents were unknown and a combined exclusion probability of 0.9968 in identity analysis to distinguish between two randomly selected individuals. These results were similar to those obtained for *A. antennatus* but with 21 loci (0.9786 and 0.9999, respectively) [53], proving the efficacy of these loci for future parentage studies in *A. foliacea*. Furthermore, our results indicate the robustness of the developed panel of microsatellites for application to future studies of genetic diversity and population structure in natural populations of the species.

## 4. Conclusions

The genomic analysis on *A. foliacea*, obtained through NGS, has enabled the validation and characterization of nine polymorphic microsatellite loci. This larger set of loci will provide a stronger set of tools to (i) perform parentage studies and (ii) examine connectivity patterns (horizontal and vertical), including examining the population structure of this species at a variety of geographical scales and, particularly, between exploited populations in shallow waters and deeper unexploited populations. Valuable information for comprehensive management policies of this exploited marine resource will be provided by integrating these results into the species’ biological knowledge.

## Figures and Tables

**Table 1:** Polymorphic microsatellite loci detected in *Aristaeomorpha foliacea*.

Locus	Repeat Motif	Primer Sequences 5′–3′	Ta (°C) ^1^	*N*_A_ ^1^	Allele Size Range (bp)
Af1a	(TA)_12_	F: GCATTCACAGCAGGAGCATA R: ACGGACATCGTCGTCATACA	60	2	177, 179
Af1b	(TAG)_7_	F: CCTGCATGAATGACGACAAC R: CCACGCAGGATTAGATCCAC	50	3	254–260
Af1c	(ATA)_7_	F: GGGAATCCCTGTTGGTTTCT R: CCTTCCTCGGTGACGTTAAA	60	2	204, 207
Af1d	(TGAC)_12_	F: GAAGGCCCGGGTAAGTTTGA R: AGCCAGTTAGCAGGAGCAAG	60	5	370–390
Af1e	(TA)_11_	F: GCCGTATATCGGCCTGTACT R: CCTCCTCCTTCTTCTTCTTGG	50	3	245–255
Af22a	(CTA)_6_ + (TGC)_8_	F: GGAGGTTTTATCCACCAGACG R: CCCAGAGAGTTCGAAAACCTT	60	6	266–281
Af22b	(CTC)_6_ + (TAA)_6_	F: TGAGCTGGGCATCTCTCTCT R: TGTTGTAGTAGCCGCCCTCT	60	5	161–176
Af22c	(TAC)_5_	F: AAATTCTCCCCCTCCTTTCC R: GGGTTTGCCTCGGAATAAAT	60	2	268, 271
Af22d	(AG)_9_	F: CGGAGACTACTCCAGCCTTG R: GTCGCGCATCGGTCTAGATA	60	5	244–252
Af40a	(TCCC)_5_	F: CCGTTCTTTCCCTCCCATA R: TTCGAATGACAACAATAATGTGC	60	3	222–234
Af40b	(TC)_8_	F: TCCTCAAGCACATTATTGTTGTCA R: AGGAAGGAACGAACGAAGGG	60	3	345–353
Af75	(AT)_10_	F: ATGGTCTGGTGTGGGTTGTT R: TAGAGCCACCAAGTGCTCCT	60	4	224–240
Af181	(AT)_29_	F: CGATGTGGGTGTGTCAAGTC R: GGCATGAATTGGTAAGTGGAG	60	2	441, 443
Af341	(TCT)_8_	F: CACCCTTCCGCTACCTCAT R: GTAGGCGTGAGAGGGTCGT	50	2	169, 172
Af518	(AGAT)_7_	F: TCAGCTGCTTTGTGAGAAGG R: CCAACGTTTAAGAGAGGCATAA	50	2	199, 203
Af880	(GTT)_7_	F: CGTCTTGACCCAAGGCTTTA R: GCTCCCACTTGCTCAAGAAC	60	2	251, 254
Af1110	(AT)_5_	F: TTGGACACGAAACCGTATCA R: CAAACTGCCGGTATCCTCTC	60	2	207, 211
Af1148	(ATA)_8_	F: TTGTTGTTGCGACACAAGAAAT R: TGATAGTGTCCCTCACAACACAC	50	2	208, 211
Af1793	(TGG)_5_	F: GGGGTAACGTCGGTTTACATT R: CCTTAACCTCGTCATTACCAACC	60	3	72–78

^1^ Ta, annealing temperature; *N*_A_, number of alleles.

**Table 2:** Characterization of 12 microsatellite loci of *A. foliacea* in a locality from the Western Mediterranean Sea.

Locus	GenBank ^1^	*N*_A_ ^1^	Allele Size Range (bp)	*H*_o_ ^1^	*H*_e_ ^1^	*F*_IS_ ^1^	*p*-Value ^1^
Af1a	PQ073196	1	177	0.000	0.000	-	-
Af1b	PQ073196	3	254–260	0.500	0.543	0.078	0.335
Af1e	PQ073196	1	245	0.000	0.000	-	-
Af22c	PQ073197	3	268–274	0.557	0.448	−0.264	0.345
Af40a	PQ073198	4	222–234	0.300	0.508	0.410	0.007
Af40b	PQ073198	10	331–355	0.433	0.851	0.491	0.000 *
Af75	PQ073199	3	234–242	0.067	0.066	−0.009	1.000
Af518	PQ073200	3	191–203	0.100	0.159	0.370	0.165
Af880	PQ073201	3	245–251	0.233	0.215	−0.086	1.000
Af1110	PQ073202	3	207–211	0.167	0.214	0.220	0.325
Af1148	PQ073203	4	205–214	0.300	0.364	0.177	0.040
Af1793	PQ073204	2	72,78	0.100	0.097	−0.036	1.000

^1^ GenBank accession number of the individual from Cabrera sequenced by NGS; *N*_A_, number of alleles; *H*_o_, observed heterozygosity; *H*_e_, expected heterozygosity; *F*_IS_, inbreeding coefficient; HWE-*p*-value. * Significant departure from HWE.

## Data Availability

The original contributions presented in the study are included in the article; further inquiries can be directed to the corresponding author.
